# Septic Arthritis With Superimposed Acute Gouty Arthritis in a Rheumatoid Arthritis Patient

**DOI:** 10.7759/cureus.24352

**Published:** 2022-04-21

**Authors:** Richard Medina-Perez, Shadi A Baajour, Sheyla Gonzalez, Jose L Batista, Daniel J Campbell

**Affiliations:** 1 Internal Medicine Residency, Aventura Hospital and Medical Center, Aventura, USA; 2 Internal Medicine, Nova Southeastern University Dr. Kiran C. Patel College of Allopathic Medicine, Fort Lauderdale, USA

**Keywords:** crystal arthritis, gout crystals, group b streptococcus agalactiae bacteremia, infectious arthritis, ortho surgery, rheumatoid arthritis

## Abstract

Septic arthritis is a rare but serious complication of both rheumatoid and gouty arthritis and can lead to significant morbidity and even mortality. Here, we report a case of septic arthritis with bacteremia, monosodium urate crystals, and hyperuricemia in a 75-year-old male with long-standing rheumatoid arthritis. Arthrocentesis revealed gram-positive cocci representing group B streptococcus (*Streptococcus agalactiae*) infection and monosodium urate crystals. A diagnosis of septic arthritis with superimposed acute gouty arthritis was made and the patient was treated accordingly. Management included surgical irrigation and debridement, antibiotic therapy, and systemic glucocorticoids which resulted in a significant improvement in the patient’s clinical status.

## Introduction

Septic arthritis is defined as an infection in the synovial fluid and tissue of a joint. While usually caused by bacterial invasion, the infection may also be due to viral, fungal, or mycobacterial microbes. When left untreated, septic arthritis can lead to a significant destruction of bone, cartilage, and surrounding soft tissue within days [1].

Rheumatoid arthritis is a systematic, inflammatory disease affecting 0.41% to 0.54% of the population in the United States [2]. Both rheumatoid arthritis and the need for frequent intra-articular glucocorticoid injections significantly increase the risk of septic arthritis in patients due to abnormal joint structure and possible direct inoculation of bacteria into the joint space during the procedure, respectively [3,4].

A diagnosis of gout can be made with serum hyperuricemia and clinical manifestations of the disease, such as recurrent flares of inflammatory arthritis, chronic arthropathy, urate crystal accumulation, or uric nephrolithiasis. Cases of gouty arthritis with concomitant septic arthritis have been documented but are rare [5,6]. Here, we report a case of septic arthritis with superimposed gouty arthritis in a patient with a history of rheumatoid arthritis.

## Case presentation

A 75-year-old male with a medical history of rheumatoid arthritis and gout presented to the emergency department with a two-day history of right knee pain, swelling, redness, decreased range of motion, and subjective chills and fevers. The patient described the pain as constant, stabbing, 10/10 in severity, and exacerbated by movement and weight-bearing. The patient denied a history of trauma to the right knee and stated that the current symptoms were different from previous rheumatic flares. The patient had presented to an outpatient rheumatology clinic three weeks prior for right knee pain and was treated with oral prednisone and an intra-articular glucocorticoid injection. Prior management of the patient’s rheumatoid arthritis included abatacept injections every two weeks. Additional medical history included hypertension managed with amlodipine 5 mg once per day and Alzheimer’s dementia managed with donepezil 5 mg once per day.

On arrival, the patient was tachycardic to 129 beats per minute, hypertensive to 161/80 mmHg, and febrile with a temperature of 103°F. On physical examination, the right knee was edematous, erythematous, and diffusely tender to palpation. The active and passive range of motion of the knee joint was limited due to pain. Lower extremity distal pulses were intact bilaterally, and there were no focal neurologic deficits. There was tenderness to palpation in the bilateral shoulders, elbows, wrists, and ankles with associated decreased range of motion. Bilateral ulnar deviation of the wrists and proximal interphalangeal and metacarpophalangeal joint edema was present. Rheumatoid nodules or tophi were not noted. The rest of the physical examination was noncontributory. Upon admission, the patient’s blood was drawn and sent for laboratory analysis (Table 1).

**Table 1:** Laboratory values. COVID-19: coronavirus disease 2019

Laboratory parameter	Patient value	Reference range
Hemoglobin, blood	14.6 g/dL	Male: 13.5–17.5 g/dL
Leukocyte count	16,100 cells/mm^3^	4,500–11,000 cells/mm^3^
Segmented neutrophils	85%	54–62%
Erythrocyte sedimentation rate	39 mm/hour	0115 mm/hour
C-reactive protein	39 mg/dL	0.811.0 mg/dL
Creatine kinase	638 U/L	Male: 25–90 U/L
Blood urea nitrogen	28 mg/dL	7–18 mg/dL
Creatinine	1.8 mg/dL	0.6–1.2 mg/dL
Uric acid	9.0 mg/dL	3.0–8.2 mg/dL
COVID-19	Negative	Negative

**Figure 1:**
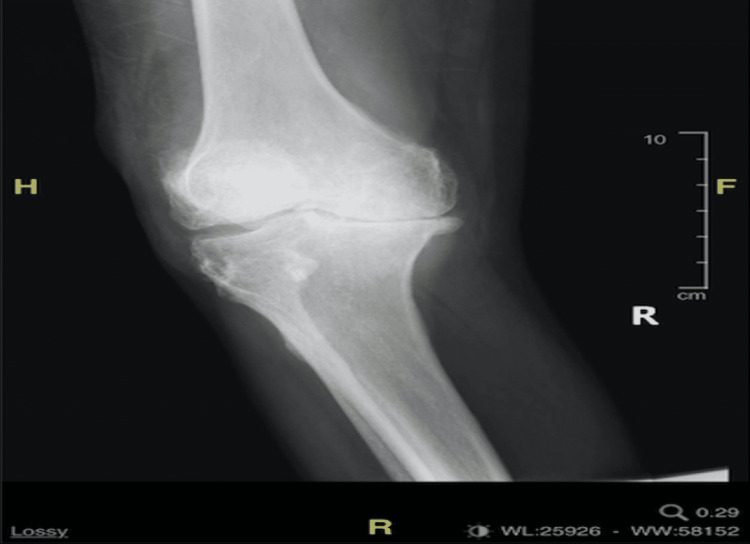
Anteroposterior radiograph of the right knee.

Imaging studies included an anteroposterior (AP) right knee radiograph demonstrating fullness in the suprapatellar bursa from joint effusion, advanced joint space narrowing of the medial compartment and patellofemoral joint with tricompartmental osteoarthritis, arteriovascular calcifications, prominent spurring of the proximal fibula and medial joint line of the knee, and spurring of the patellofemoral joint, anterior and posterior tibia, and femur (Figure 1). Unfortunately, the lateral view was not available. An AP chest radiograph was also performed which demonstrated low lung volumes and bibasilar plate-like atelectasis.

Venous duplex ultrasound of the bilateral lower extremities revealed no evidence of deep venous thrombosis.

Arthrocentesis of the right knee was performed. Synovial fluid analysis revealed a total leukocyte count of 65.0 cells/L with polymorphonucleocyte predominance and positive monosodium urate crystals, which according to the diagnostic criteria presented by the American Academy of Family Physicians and the American College of Rheumatology confirms a diagnosis of acute septic and gouty arthritis [7,8]. Synovial fluid gram stain was significant for 1+ white blood cells present with no stainable organisms. Synovial fluid culture and blood culture revealed gram-positive cocci in pairs suggestive of *Streptococcus agalactiae*, group B.

At this time, Infectious Disease was consulted that recommended an antibiotic course consisting of Rocephin intravenous (IV) 2 g per day for a duration of four weeks. An echocardiogram was performed which revealed no acute valvular pathology to indicate bacterial endocarditis. After no improvement in the patient’s clinical condition and physical examination findings by hospital day three, Orthopedic Surgery was consulted. Uncomplicated arthroscopic irrigation and debridement of the right knee was recommended and subsequently performed. The patient’s clinical status improved, and the patient was discharged to a skilled nursing facility on hospital day nine.

## Discussion

Our case report highlights the importance of the prompt diagnosis of septic arthritis and the role of a multidisciplinary team in the management of concomitant septic and gouty arthritis in rheumatic patients.

Studies have shown a higher incidence of infection in individuals with rheumatoid arthritis due to increased exposure to exogenous steroids [3] and the use of intraarticular glucocorticoid injections [9]. However, the combination of septic and gouty arthritis in a rheumatic patient is rare. One study found that 43.5% of patients with septic arthritis also had evidence of gouty arthritis, but none of the patients had a history of rheumatoid arthritis [10].

The management of a septic joint with superimposed gouty arthritis in a rheumatic patient involves balancing antibiotic coverage, anti-inflammatory therapies, and immunosuppressive medications. Due to our patient’s decreased kidney function, he could not receive non-steroidal anti-inflammatory drugs or colchicine, the treatment choice for an acute gout flare. As a result of the patient’s lack of improvement with antibiotics, irrigation and debridement were necessary. Surgical drainage allows the removal of debris such as polymorphonuclear byproducts and bacterial toxins, break up of loculations, and an opportunity to place irrigation-suction systems [11]. Surgical irrigation and debridement have been proven to be more effective than repeated aspiration [12,13]. Goldstein et al. treated rabbits with *Staphylococcus aureus* pyogenic arthritis with antibiotics and either serial aspirations or arthrotomy and irrigation. The animals treated with aspiration had worse outcomes evidenced by a greater degree of thinning cartilage, acellularity, and cloning of chondrocytes [12]. Additionally, Bynum et al. performed a 10-year retrospective study of 46 joints affected by acute hematogenous pyogenic arthritis. They found that needle aspiration was correlated with increased morbidity and mortality. Seven knees with poor results, judged by the level of pain and range of motion at follow-up, were treated with aspirations. The two patients who died in their series were treated with aspirations [13]. Bynum et al. also postulated that patients with underlying diseases, such as rheumatoid arthritis or immunosuppression, should be treated with surgical drainage to prevent septicemia or aid in its treatment if already present [13].

Better outpatient management of this patient’s inflammatory arthropathy and comorbid conditions could have prevented his septicemia and his inpatient hospital stay. Once admitted, the collaboration between Internal Medicine, Rheumatology, Infectious Disease, and Orthopedic Surgery, along with ancillary staff, was crucial in optimizing the patient’s condition. Studies have shown that the use of multidisciplinary teams improves patient outcomes, reduces adverse events, and increases patient and staff satisfaction [14]. Patient safety is improved when a team of providers discusses care rather than individually making recommendations [15]. Lamb et al. found that recommendations formulated by a collaborative team of experts will differ up to 50% from those created by multiple independent providers [16]. Thus, the collective power of a team approach to patient care can be argued to be superior to an individualistic approach.

## Conclusions

This case highlights the management of septic arthritis in a patient with significant comorbid risk factors for infection and complicated pathologic processes. A background of rheumatic joint disease, direct inoculation, and chronic immunosuppressive medications played a role in leading to an infection that did not respond to traditional antibiotics. We felt it necessary to proceed with arthroscopic irrigation and debridement which resulted in an improvement in the patient’s condition.
